# The prognostic role of survivin expression in breast cancer: A systematic review and meta-analysis

**DOI:** 10.1097/MD.0000000000040013

**Published:** 2024-10-04

**Authors:** Betul Bolat Kucukzeybek, Yuksel Kucukzeybek, Yasemin Basbinar, Hulya Ellidokuz, Mustafa Agah Tekindal, Cigdem Dinckal, Mustafa Oktay Tarhan

**Affiliations:** a Department of Pathology, Izmir Katip Celebi University Ataturk Training and Research Hospital, Izmir, Turkey; b Department of Medical Oncology, Izmir Katip Celebi University Ataturk Training and Research Hospital, Izmir, Turkey; c Dokuz Eylul University, Institute of Oncology, Izmir, Turkey; d Department of Biostatistics, Izmir Katip Celebi University, Izmir, Turkey.

**Keywords:** BIRC5, breast cancer, survivin

## Abstract

**Background:**

Breast cancer is a heterogeneous condition with variations in histopathological, genomic, and biological characteristics. Although clinicopathological prognostic factors and gene expression profiles are commonly used to guide treatment decisions in patients with breast cancer, there is still a need for new prognostic markers. One potential marker is survivin, a protein belonging to the apoptosis inhibitor family. However, studies examining the relationship between survivin and prognosis in breast cancer have yielded inconsistent results. This study aimed to evaluate the impact of survivin expression on the prognosis of breast cancer patients through a meta-analysis.

**Methods:**

Studies evaluating survivin expression were sourced from the PubMed, Embase, and Cochrane databases. We conducted a meta-analysis based on full-text articles that evaluated the relationship between survivin expression and survival by immunochemistry or polymerase chain reaction. The studies were initially divided into 2 groups based on the evaluation of overall survival (OS) and disease-free survival (DFS). Subsequently, each group was further categorized according to the method used to detect survivin expression. Statistical analyses for this study were conducted using Stata and JAMOVI.

**Results:**

After screening with keywords, we identified 24 retrospective studies evaluating OS and 15 retrospective studies evaluating DFS, which were included in the analysis. We found that the studies in the meta-analysis were not heterogeneous, and this remained consistent when categorizing the groups by survivin expression detection. Survivin expression was associated with OS (HR 1.23, 95% CI 0.81–1.65) and DFS (HR 0.89, CI 0.42–1.36), indicating poor prognosis. This significant relationship between survivin expression and survival persisted when the studies were categorized by the detection method, either immunohistochemistry or polymerase chain reaction.

**Conclusion:**

In this study, we evaluated the prognostic significance of survivin expression in patients with breast cancer through a meta-analysis. These results support the use of survivin expression as a prognostic marker in breast cancer, potentially guiding treatment decisions.

## 1. Introduction

Breast cancer ranks first in incidence among women and is a leading cause of cancer-related deaths.^[1]^ It is a highly heterogeneous disease in terms of clinical and molecular characteristics, biological behavior, and response to treatments. Despite individualized surgical treatments, radiotherapy, and systemic therapies guided by gene expression panels or immunohistochemical subgroups, recurrence can occur, often leading to poor prognosis.^[2]^ While widespread adjuvant systemic therapy has reduced breast cancer mortality rates in the Western world, many patients still do not receive appropriate treatment. Some patients receive systemic treatment when local treatment is sufficient, whereas others do not receive adequate or appropriate systemic therapy. Reliable prognostic factors that may help identify patients at the highest risk of recurrence and clinically usable predictive factors may help personalize treatment. All of these could potentially protect patients from exposure to any possible treatment toxicity.^[3]^ Ozmen et al found that the median age at diagnosis of breast cancer was 51, and the majority of patients were at stage 2 at the time of diagnosis in Turkey. Histopathology was invasive ductal cancer in 77%. During the mean 51.6 months of follow-up, the local/regional and systemic recurrence rates were 3.7% and 5.2%, respectively; 5 and 10-year overall survival (OS) rates were 86% and 76%.^[4]^

Carcinogenesis is associated with an imbalance between apoptosis and cell proliferation. Various genes are either pro-apoptotic or antiapoptotic and regulate apoptotic signaling pathways. Among the proteins that inhibit apoptosis, survivin is a key player.^[5]^ Survivin is a multifunctional protein involved in the control of cell proliferation, inhibition of apoptosis, and promotion of angiogenesis. It is highly expressed in tumor tissues, but it is either undetectable or expressed at very low levels in normal tissues. Survivin is crucial for normal cell division and is involved in chromosomal segregation and cytokinesis. Its increased expression in tumor cells can disrupt normal cell cycle control, allowing cells with microtubule defects or abnormal chromosome segregation to proceed through division. Additionally, mitochondrial survivin can block apoptosis independently of the cell cycle.^[6,7]^ Increased survivin expression is associated with poor prognosis in various cancers, including hematological malignancies; lung, colon, bladder, ovarian, prostate, hepatocellular, pancreatic, and esophageal cancers; and neuroblastoma.^[8–17]^ However, its role as a prognostic factor for breast cancer remains controversial. Studies have shown conflicting results, with survivin expression being associated with poor prognosis, good prognosis, or no association with prognosis in patients with breast cancer patients.^[18–21]^ In patients diagnosed with breast cancer, survivin expression is typically assessed by immunohistochemistry (IHC) or polymerase chain reaction (PCR) methods.

There remains a need for new prognostic factors to inform treatment decisions for patients with breast cancer. We conducted a meta-analysis to evaluate the contribution of survivin, a member of the family of apoptosis inhibitor proteins, to the prognosis of patients with breast cancer. Our aim was to summarize the prognostic significance of survivin expression in breast cancer patients, considering the method of survivin expression evaluation based on literature data, and to provide a prognostic marker that can guide treatment decisions and help the decision-making process for the treatment of breast cancer.

## 2. Materials and methods

### 2.1. Literature search

Data were collected from PubMed, Embase, and Cochrane databases, with a literature search conducted until January 2024. For studies evaluating the association between survivin expression and breast cancer survival, we used the following key search MESH terms: “”breast cancer“ and ” survivin’’.

### 2.2. Data extraction

The titles and abstracts of the articles were reviewed, and irrelevant articles were eliminated. The meta-analysis was based on the full texts of the remaining studies and the selection of appropriate publications according to the inclusion and exclusion criteria. The collected data included the name of the first author, year of publication, country, study type, number of patients, histological subtypes of breast cancer, disease stage, OS, disease-free survival (DFS), detection method for survivin expression, hazard ratios, and confidence intervals. The effects of survivin expression on OS and DFS were calculated using hazard ratios and confidence intervals.

### 2.3. Inclusion and exclusion criteria

The meta-analysis included studies evaluating the relationship between survivin expression and OS in patients diagnosed with breast cancer, studies evaluating the relationship between survivin expression and DFS in patients diagnosed with breast cancer, studies reporting survival results and hazard ratios or data suitable for calculation, and studies assessing survivin expression by IHC or PCR. Exclusion criteria were case reports, case series, reviews, letters to the editor, studies with unavailable full text, and studies that used methods other than IHC or PCR to determine survivin expression.

### 2.4. Statistical analysis and quality assessment

Statistical analyses for this study were conducted using Stata (STATA Corporation, College Station Texas; www.stata.com) and JAMOVI (The jamovi Project 2023 jamovi (Version 2.3) [Computer Software]) (https://www.jamovi.org). A *P*-value threshold of <.05 was set for statistical significance. The quality of the studies included in the meta-analysis was assessed using the Newcastle–Ottawa Quality Assessment Scale Cohort Studies, with studies scoring 0 to 3 classified as low quality, 4 to 6 as medium quality, and 7 to 9 as high quality.^[22]^

The meta-analysis included studies that examined the relationship between survivin expression and OS in breast cancer patients, as well as those that evaluated the relationship between survivin expression and DFS. Each study was categorized based on the method used to evaluate survivin expression, either by IHC or PCR. Analyses were performed generally for the DFS and OS groups and in more detail according to the evaluation method.

Egger Linear Regression Test was used to determine if the effect sizes and standard errors of the studies in the meta-analysis were linear. Duval and Tweedie Trim and Fill method was applied to reduce publication bias, and the common exposure value was recalculated. The random-effects model (Paule–Mandel method) was used to detect within-study and between-study variances. The heterogeneity of the effect sizes was evaluated using Cochrane *Q* statistic with degrees of freedom (k − 1), the I² statistic to determine the level of heterogeneity, and the τ² statistic to determine the true variance between studies.

Fail-Safe N analysis was used to assess publication bias and the effect of a small sample size. Funnel plot asymmetry was examined using the Rank Correlation Test, and the Regression Test was applied for a statistical evaluation of funnel plot asymmetry.

Our meta-analysis used a methodological approach that incorporated a wide range of statistical analyses. Various statistical tests and models were used to ensure the accuracy and reliability of effect sizes. The random-effects model provided more accurate and generalizable results by accounting for heterogeneity and variance across different studies. Tests such as Egger Linear Regression Test and the Rank Correlation Test further enhanced the reliability of our findings by assessing potential publication bias and small sample size effects.

Ethics committee approval was obtained from the İzmir Dokuz Eylül University Non-Interventional Research Ethics Committee.

### 2.5. Conflict of interest

The authors declare that they have no competing interests.

## 3. Results

**Figure 1.**
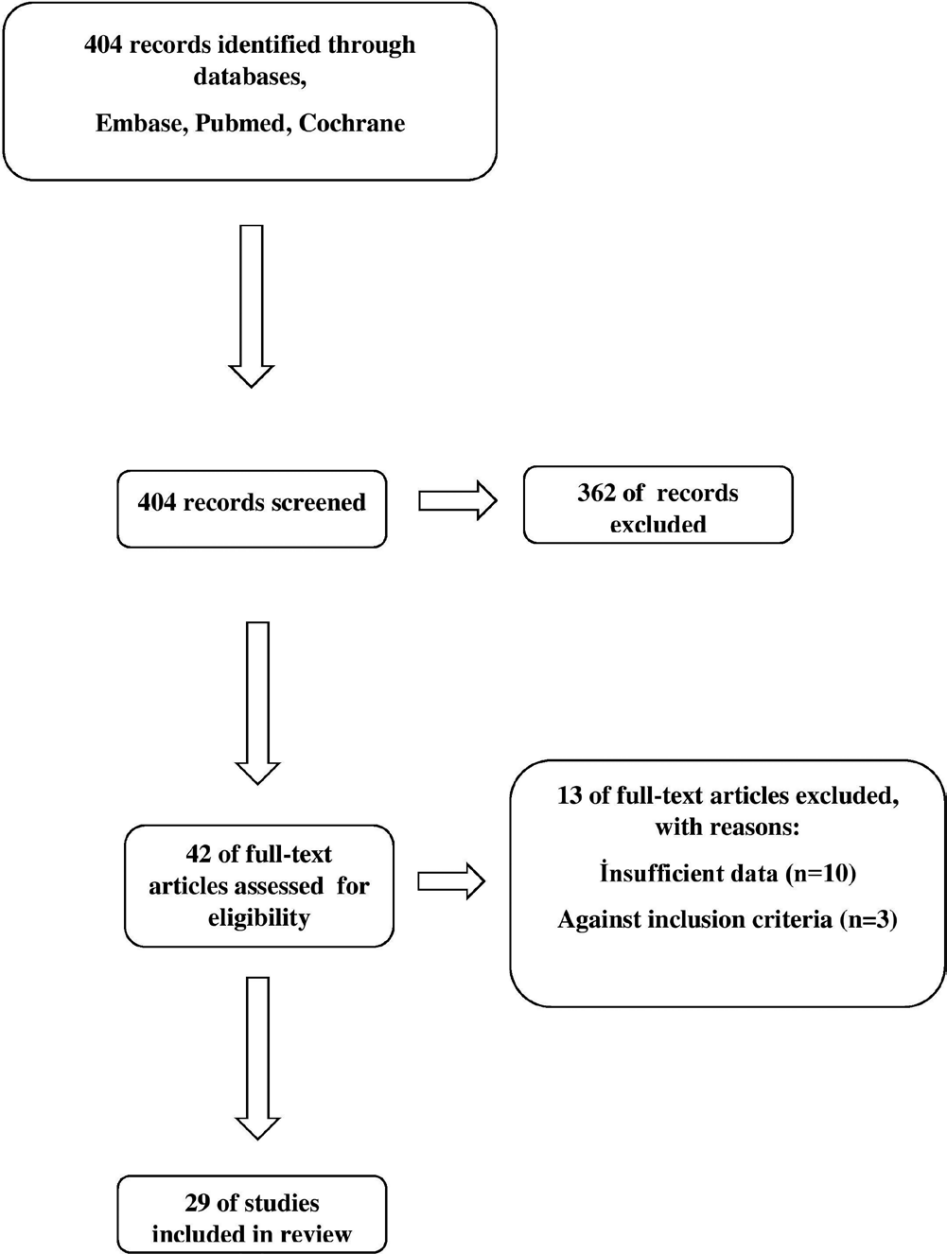
Literature screening flow chart.

After searching databases using the keywords “survivin” and “breast cancer, ” we identified 404 articles. The literature screening flow chart is shown in Figure 1. The main features of the included studies are shown in Table 1: study site, data collection, number of patients, main outcome, method, tumor type, and stage. Our meta-analysis included 24 studies (n = 9306) evaluating the relationship between survivin expression and OS and 15 studies (n = 9083) evaluating the relationship between survivin expression and DFS in breast cancer. In addition, when categorizing studies by the method used to determine survivin expression, the meta-analysis included 19 studies (n = 5154) evaluating OS using IHC, 5 studies (n = 4076) evaluating OS using PCR, 7 studies (n = 2643) evaluating DFS using IHC, and 8 studies (n = 6440) evaluating DFS using PCR. All included studies retrospectively examined survivin expression in patient groups with varying histologic subtypes and different disease stages. This variability may have contributed to an intermediate effect size in the meta-analysis results owing to the lack of standardization in the evaluation methods.

**Table 1:** The main features of the included studies.

Author, year of publication	Country	Data collection	Number of patients	Main outcome	Method	Tumor type	Stage
Adinew G.M et al (2022)	USA	Retrospective	1879	OS, DFS	IHC	All	All
Al-Joudi FS et al (2007)	Malaysia	Retrospective	170	OS	IHC	All	
Athanassiadou AM et al (2011)	Greece	Retrospective	140	OS	IHC	All	1, 2, 3
Brennan DJ et al (2008)	Ireland	Retrospective	102	OS	IHC	All	1, 2, 3
Cao Q et al (2023)	China	Retrospective	947	OS	IHC	All	All
Dedić Plavetić N et al (2013)	Croatia	Retrospective	215	OS,DFS	IHC	All	2, 3
Dogu GG et al (2010)	Turkey	Retrospective	30	OS,DFS	IHC	TNBC	1, 2, 3
Hamy AS et al (2016)	France	Retrospective	188	DFS	PCR	All	2, 3
Ionta MT et al (2010)	Italy	Retrospective	53	OS	IHC	All	3
Kennedy SM et al (2003)	Ireland	Retrospective	293	OS,DFS	IHC	All	1, 2, 3
Kleinberg L et al (2007)	Norway	Retrospective	47	DFS	IHC	All	All
Kostadima L et al (2006)	Greece	Retrospective	272	OS,DFS	PCR	All	2, 3
Li S et al (2017)	China	Retrospective	110	OS	IHC	All	1, 2, 3
Liu N et al (TNBC) (2022)	China	Retrospective	90	OS,DFS	IHC	TNBC	2, 3
Liu N et al (non-TNBC) (2022)	China	Retrospective	52	OS,DFS	IHC	Non-TNBC	2, 3
Massidda B et al (2010)	Italy	Retrospective	53	OS	IHC	All	2, 3
Mehraj U et al (2022)	India	Retrospective	4929	DFS	PCR		
Nassar A et al (2008)	USA	Retrospective	37	OS,DFS	IHC	All	All
O’Driscoll L et al (2003)	Ireland	Retrospective	106	OS,DFS	PCR	All	1, 2, 3
Oparina N et al (2021)	Sweden	Retrospective	3273	OS	PCR		All
Rexhepaj E et al (2010)	Ireland	Retrospective	359	OS	IHC	All	1, 2, 3
Shi CT et al (2019)	China	Retrospective	150	OS,DFS	PCR	TNBC	1, 2, 3
Sohn DM et al (2006)	Korea	Retrospective	80	OS	IHC	All	All
Span PN et al (2004)	Netherlands	Retrospective	275	OS,DFS	PCR	All	1, 2, 3
Span PN et al (2006)	Netherlands	Retrospective	275	DFS	PCR	All	1, 2, 3
Tanaka et al (2000)	Japan	Retrospective	167	OS	IHC	All	1, 2, 3
Tsai WC et al (2008)	Taiwan	Retrospective	151	OS	IHC	All	All
Xu C et al (2014)	Japan	Retrospective	245	DFS	PCR	All	1, 2, 3
Yakirevich E et al (2012)	USA	Retrospective	226	OS	IHC	All	All
Yamashita S et al (2007)	Japan	Retrospective	76	OS	PCR	All	1, 2, 3

DFS = disease-free survival, IHC = immunohistochemistry, OS = overall survival, PCR = polymerase chain reaction, TNBC = triple-negative breast cancer.

The publication quality of the studies was assessed and is presented in Table 2 (OS studies) and Table 3 (DFS studies). All of the studies evaluating OS and DFS included in the meta-analysis were found to be medium or high-quality articles.

**Table 2:** Evaluation of publication quality for overall survival.

Study	Representatives of the exposed cohort	Selection of the nonexposed cohort	Ascertainment of exposure	Demonstration that outcome of interest was not present at start of study	Comparability of cohorts on the basis of the design or analysis	Assessment of outcome	Was follow-up long enough for outcomes to occur?	Adequacy of follow-up of cohorts	Total
Adinew GM (2022)	C (0)	A (+1)	A (+1)	A (+1)	B (+1)	A (+1)	A (+1)	A (+1)	7
Al-Joudi FS (2007)	C (0)	A (+1)	A (+1)	A (+1)	B (+1)	A (+1)	A (+1)	A (+1)	7
Athanassiadou AM (2011)	C (0)	B (0)	A (+1)	A (+1)	B (+1)	A (+1)	A (+1)	A (+1)	6
Brennan DJ (2008)	C (0)	A (+1)	A (+1)	A (+1)	B (+1)	A (+1)	A (+1)	A (+1)	7
Cao Q (a) (2023)	C (0)	A (+1)	A (+1)	A (+1)	B (+1)	A (+1)	A (+1)	A (+1)	7
Dogu GG (2010)	C (0)	A (+1)	A (+1)	A (+1)	B (+1)	A (+1)	B (0)	D (0)	5
Ionta MT (2010)	C (0)	A (+1)	A (+1)	A (+1)	B (+1)	A (+1)	A (+1)	A (+1)	7
Kennedy SM (2003)	C (0)	A (+1)	A (+1)	A (+1)	B (+1)	A (+1)	A (+1)	D (0)	6
Kostadima L (2006)	C (0)	A (+1)	A (+1)	A (+1)	B (+1)	A (+1)	A (+1)	A (+1)	7
Li S (2017)	C (0)	A (+1)	A (+1)	A (+1)	B (+1)	A (+1)	B (0)	D (0)	5
Liu N (TNBC) (2022)	C (0)	B (0)	A (+1)	A (+1)	B (+1)	A (+1)	A (+1)	A (+1)	6
Liu N (non-TNBC) (2022)	C (0)	A (+1)	A (+1)	A (+1)	B (+1)	A (+1)	A (+1)	A (+1)	7
Massidda B (2010)	C (0)	A (+1)	A (+1)	A (+1)	B (+1)	A (+1)	A (+1)	A (+1)	7
Nassar A (2008)	C (0)	A (+1)	A (+1)	A (+1)	B (+1)	A (+1)	B (0)	D (0)	5
O’Driscoll L (2003)	C (0)	A (+1)	A (+1)	A (+1)	B (+1)	A (+1)	A (+1)	A (+1)	7
Oparina N (2021)	C (0)	A (+1)	A (+1)	A (+1)	B (+1)	A (+1)	A (+1)	D (0)	6
Plavetic ND (2013)	C (0)	A (+1)	A (+1)	A (+1)	B (+1)	A (+1)	A (+1)	A (+1)	7
Rexhepaj E (2010)	C (0)	B (0)	A (+1)	A (+1)	B (+1)	A (+1)	B (0)	D (0)	4
Shi CT (2019)	C (0)	A (+1)	A (+1)	A (+1)	B (+1)	A (+1)	A (+1)	A (+1)	7
Sohn DM (2006)	C (0)	A (+1)	A (+1)	A (+1)	B (+1)	A (+1)	A (+1)	C (0)	6
Span PN (2004)	C (0)	A (+1)	A (+1)	A (+1)	B (+1)	A (+1)	A (+1)	A (+1)	7
Tanaka K (2000)	C (0)	A (+1)	A (+1)	A (+1)	B (+1)	A (+1)	B (0)	D (0)	5
Tsai WC (2008)	C (0)	B (0)	A (+1)	A (+1)	B (+1)	A (+1)	A (+1)	A (+1)	6
Yakirevich E (2012)	C (0)	A (+1)	A (+1)	A (+1)	B (+1)	A (+1)	A (+1)	A (+1)	7
Yamashita SI (2007)	C (0)	A (+1)	A (+1)	A (+1)	B (+1)	A (+1)	A (+1)	D (0)	6

**Table 3:** Evaluation of publication quality for disease-free survival.

Study	Representatives of the exposed cohort	Selection of the nonexposed cohort	Ascertainment of exposure	Demonstration that outcome of interest was not present at start of study	Comparability of cohorts on the basis of the design or analysis	Assessment of outcome	Was follow-up long enough for outcomes to occur?	Adequacy of follow-up of cohorts	Total
Adinew GM (2022)	C (0)	B (0)	A (+1)	A (+1)	B (+1)	A (+1)	A (+1)	A (+1)	6
Dogu GG (2010)	C (0)	A (+1)	A (+1)	A (+1)	B (+1)	A (+1)	A (+1)	A (+1)	7
Kennedy SM (2003)	C (0)	A (+1)	A (+1)	A (+1)	B (+1)	A (+1)	A (+1)	A (+1)	7
Kostadima L (2006)	C (0)	A (+1)	A (+1)	A (+1)	B (+1)	A (+1)	B (0)	D (0)	5
Liu N (TNBC) (2022)	C (0)	A (+1)	A (+1)	A (+1)	B (+1)	A (+1)	A (+1)	A (+1)	7
Liu N (non-TNBC) (2022)	C (0)	A (+1)	A (+1)	A (+1)	B (+1)	A (+1)	A (+1)	D (0)	6
Mehraj U (b) (2022)	C (0)	A (+1)	A (+1)	A (+1)	B (+1)	A (+1)	A (+1)	A (+1)	7
Nassar A (2008)	C (0)	A (+1)	A (+1)	A (+1)	B (+1)	A (+1)	A (+1)	C (0)	6
O’Driscoll L (2003)	C (0)	A (+1)	A (+1)	A (+1)	B (+1)	A (+1)	A (+1)	A (+1)	7
Plavetic ND (2013)	C (0)	A (+1)	A (+1)	A (+1)	B (+1)	A (+1)	B (0)	D (0)	5
Shi CT (2019)	C (0)	B (0)	A (+1)	A (+1)	B (+1)	A (+1)	A (+1)	A (+1)	6
Span PN (2004)	C (0)	A (+1)	A (+1)	A (+1)	B (+1)	A (+1)	A (+1)	A (+1)	7
Hamy AS (2011)	C (0)	A (+1)	A (+1)	A (+1)	B (+1)	A (+1)	A (+1)	A (+1)	7
Kleinberg L (2007)	C (0)	A (+1)	A (+1)	A (+1)	B (+1)	A (+1)	B (0)	D (0)	5
Span PN (2006)	C (0)	A (+1)	A (+1)	A (+1)	B (+1)	A (+1)	A (+1)	A (+1)	7
Xu C (2014)	C (0)	A (+1)	A (+1)	A (+1)	B (+1)	A (+1)	A (+1)	D (0)	6

Various statistical tests and models, such as Egger Linear Regression Test, Duval and Tweedie Trim and Fill method, Cochrane *Q* statistic, and I² and τ² statistics, were used in these studies. The summary statistics for publication bias are shown in Table 4. According to this table, the results for groups describing DFS with IHC and PCR methods (DFSg), DFS with IHC method (DFS-IHCg), and DFS with PCR method (DFS-PCRg), OS with IHC and PCR methods (OSg), OS with IHC method (OS-IHCg), and OS with PCR method (OS-PCRg) were reliable. The meta-analysis showed statistically significant asymmetry for the DFSg, OSg, DFS-IHCg, DFS-PCRg, OS-IHCg, and OS-PCRg groups. The I^2^ values of the studies were 0%, indicating no heterogeneity, and there was no bias in the meta-analysis results of the studies with DFSg, OSg, DFS-IHCg, DFS-PCRg, OS-IHCg, and OS-PCRg. The average effect size (Tau) was 0.000 for DFSg, OSg, DFS-IHCg, DFS-PCRg, OS-IHCg, and OS-PCRg (*P* > .001).

**Table 4:** Summarized statistics for publication bias.

	Fail-Safe N analysis (file drawer analysis)		Rank Correlation Test for Funnel plot asymmetry		Regression Test for Funnel plot asymmetry		Heterogeneity statistics						
	Fail-Safe N	*P*	Kendall Tau	*P*	Z	*P*	Tau	Tau²	I²	H²	df	Q	*P*
DFSg	16.000	<.001	0.748	<.001	2.037	.042	0.000	0 (SE. = 0.3409)	%0	1.000	15.000	4.599	.995
OSg	25.000	<.001	0.873	<.001	4.123	<.001	0.000	0 (SE. = 0.3263)	%0	1.000	24.000	18.802	.762
DFS-IHCg	8.000	<.001	0.786	.006	1.617	.106	0.000	0 (SE. = 0.4628)	%0	1.000	7.000	2.828	.900
DFS-PCRg	8.000	<.001	0.929	<.001	1.285	.199	0.000	0 (SE. = 0.5413)	%0	1.000	7.000	1.716	.974
OS-IHCg	19.000	<.001	0.836	<.001	2.828	.005	0.000	0 (SE. = 0.2667)	%0	1.000	18.000	8.355	.973
OS-PCRg	5.000	<.001	1.000	.017	0.621	.535	0.000	0 (SE. = 2.7346)	%0	1.000	4.000	0.412	.982

DFSg = disease-free survival with immunohistochemistry and polymerase chain reaction methods, DFS-IHCg = disease-free survival with immunohistochemistry method, DFS-PCRg = disease-free survival with polymerase chain reaction method, OSg = overall survival with immunohistochemistry and polymerase chain reaction methods, OS-IHCg = overall survival with immunohistochemistry method, OS-PCRg = overall survival with polymerase chain reaction method.

A random-effects model was selected for the analysis, and the statistical values are shown in Table 5. Key findings showed that the effect size for DFS was 0.890 (95% CI 0.416–1.363) (*P* < .001), indicating a significant effect of the methods over the period 2003–2022 and a positive relationship with DFS. The effect size for OSg was 1.230 (95% CI 0.814–1.647) (*P* < .001), indicating a significant effect of the methods over the period 2000 to 2023 and a positive relationship with OSg. The effect size for DFS-IHCg was 0.890 (95% CI 0.416–1.363) (*P* < .001), indicating a significant effect of the methods over the period 2003 to 2022 and a positive relationship with DFS-IHCg. The effect size for DFS-PCRg was 0.951 (95% CI 0.254–1.649) (*P* < .001), indicating a significant effect over the period 2003 to 2022, and a positive relationship with DFS-PCRg. The effect size for OS-IHCg was 0.787 (95% CI 0.383–1.189) (*P* < .001), indicating a significant effect of the methods over the period 2000 to 2023, and a positive relationship with OS-IHCg. The effect size for OS-PCRg was 1.240 (95% CI −0.482 to 2.966) (*P* = .158), suggesting that the methods may not have a significant effect over the specified period (2003–2021), and OS-PCRg may not have a positive relationship with the examined situation. These results should be interpreted with caution.

**Table 5:** Statistics for random-effects model.

	Random-effects model						
		Estimate	SE	Z	*P*	CI lower bound	CI upper bound
DFSg	Intercept	0.890	0.242	3.68	<.001	0.416	1.363
OSg	Intercept	1.230	0.213	5.79	<.001	0.814	1.647
DFS-IHCg	Intercept	0.890	0.242	3.68	<.001	0.416	1.363
DFS-PCRg	Intercept	0.951	0.356	2.67	<.001	0.254	1.649
OS-IHCg	Intercept	0.787	0.205	3.83	<.001	0.384	1.189
OS-PCRg	Intercept	1.240	0.879	1.41	.158	−0.482	2.966

DFSg = disease-free survival with immunohistochemistry and polymerase chain reaction methods, DFS-IHCg = disease-free survival with immunohistochemistry method, DFS-PCRg = disease-free survival with polymerase chain reaction method, OSg = overall survival with immunohistochemistry and polymerase chain reaction methods, OS-IHCg = overall survival with immunohistochemistry method, OS-PCRg = overall survival with polymerase chain reaction method.

### 3.1. DFS

**Figure 2.**
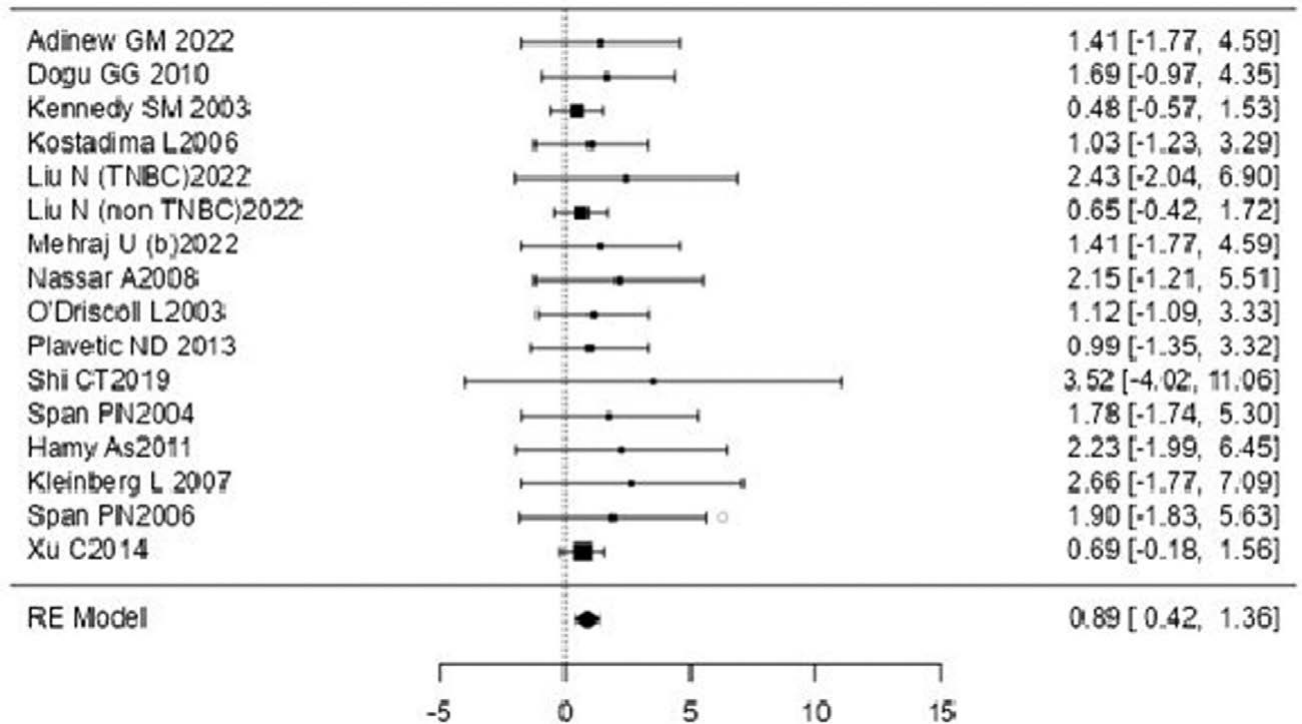
Forest plot for disease-free survival.

**Figure 3.**
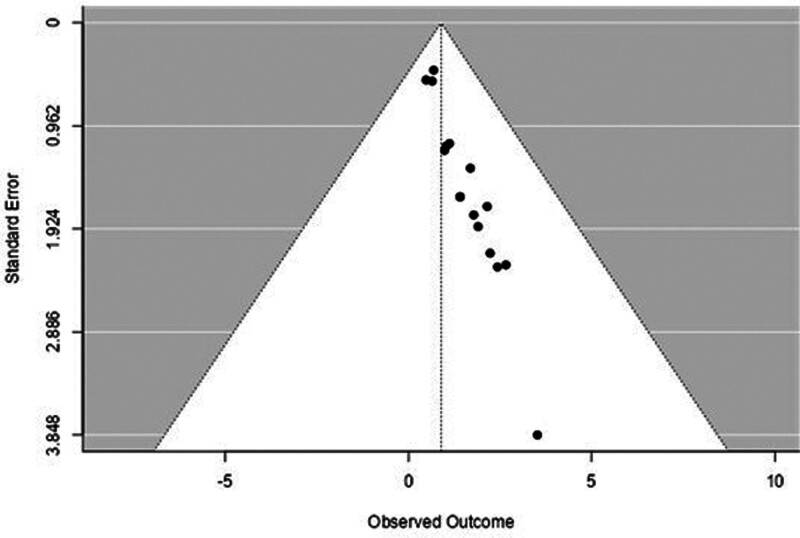
Funnel plot for disease-free survival.

A forest plot examining the association between survivin expression and DFS is shown in Figure 2. According to Figure 2, the studies with the highest weight and consistency in the meta-analysis are Xu C (2014), Kennedy SM (2003), and Liu N (non-TNBC) (2022), respectively. The study with the lowest weight and consistency was Shi CT (2019). Survivin expression was associated with lower DFS (HR 0.89, 95% CI 0.42–1.36). The funnel plot examining the relationship between survivin expression and DFS is shown in Figure 3, where the studies are concentrated at the top, indicating that the studies on DSFg included in the meta-analysis were high-power studies. Because the studies were within the confidence intervals, they had no publication bias.

### 3.2. Overall survival

**Figure 4.**
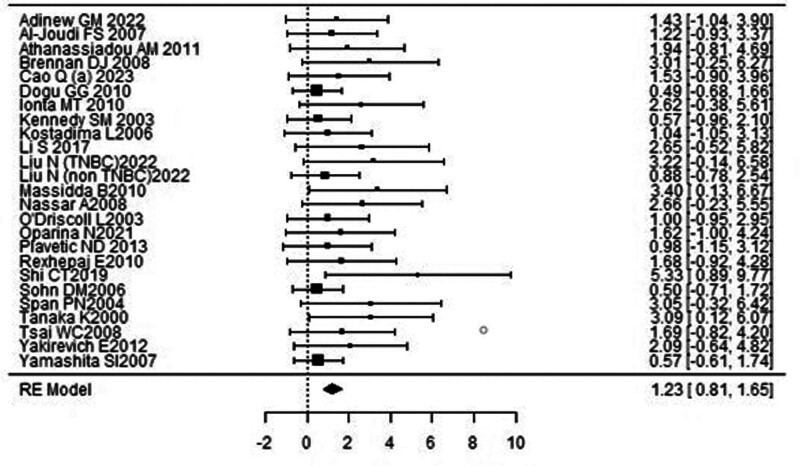
Forest plot for overall survival.

**Figure 5.**
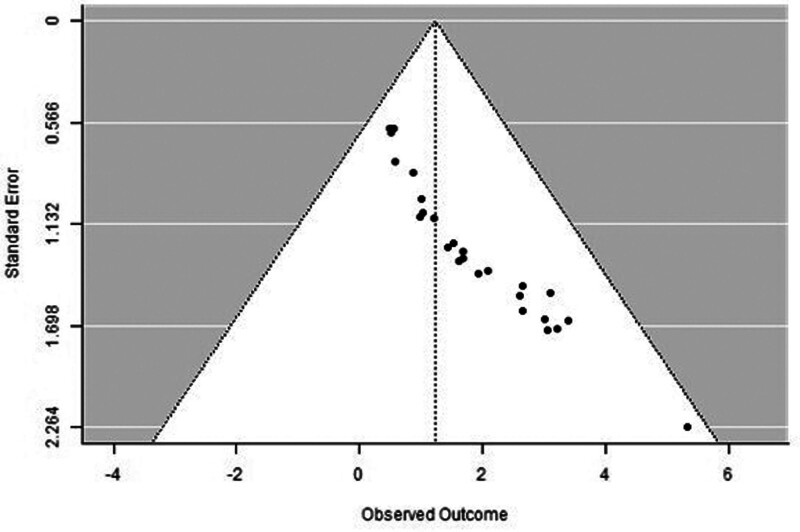
Funnel plot for overall survival.

A forest plot examining the association between survivin expression and OS is shown in Figure 4. According to Figure 4, the studies with the highest weight and consistency in the meta-analysis were Dogu GG (2010), Sohn DM (2006) and Yamashita SI (2007), respectively, while the study with the lowest weight and consistency was Shi CT (2019). Survivin expression was associated with OS (HR 1.23, 95% CI 0.81–1.65). The funnel plot evaluating the relationship between survivin expression and OS is shown in Figure 5, where the studies are concentrated at the top, indicating that the studies on OSg included in the meta-analysis were high-power studies. Because the studies were within the confidence intervals, they had no publication bias.

### 3.3. DFS (IHC)

**Figure 6.**
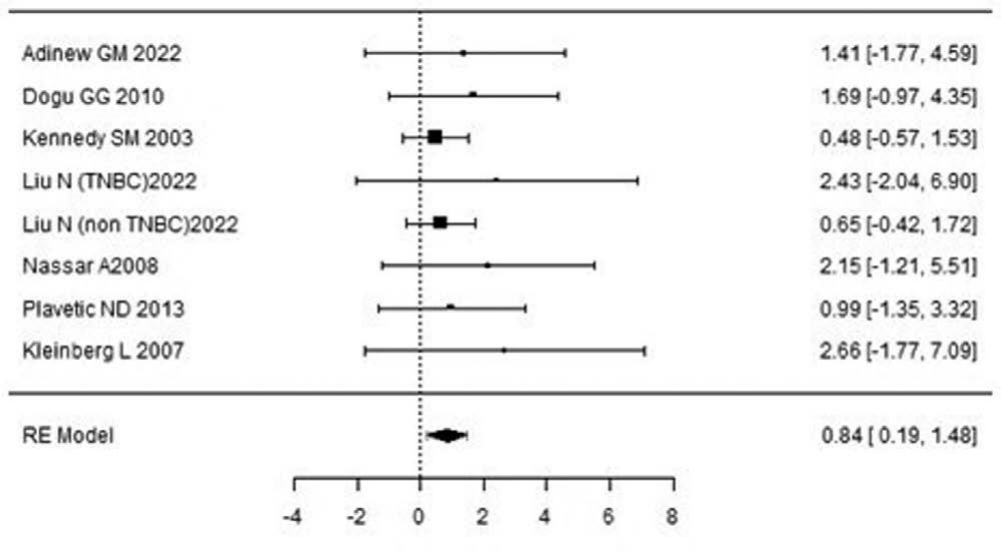
Forest plot for disease-free survival assessed by immunohistochemical method.

**Figure 7.**
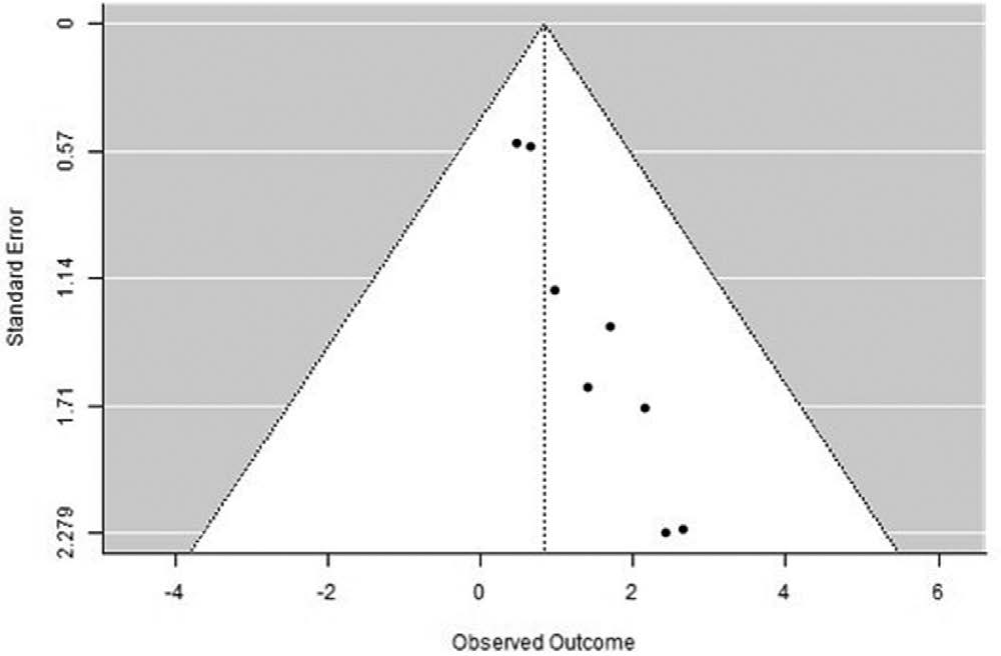
Funnel plot for disease-free survival assessed by immunohistochemical method.

The forest plot examining the association between survivin expression detected by IHC and DFS is shown in Figure 6. According to Figure 6, the studies with the highest weight and consistency in the meta-analysis were Kennedy SM (2003) and Liu N (non-TNBC) (2022), respectively while the studies with the lowest weight and consistency were Kleinberg L (2007) and Liu N (TNBC) (2022). It was shown that survivin expression evaluated by the IHC method was associated with lower DFS (HR 0.84, 95% CI 0.19–1.48). The funnel plot evaluating the relationship between survivin expression detected by IHC and DFS is shown in Figure 7, where studies are concentrated in the middle part of the graph, indicating that the studies on DFS IHC were moderately powered. Because the studies were within the confidence intervals, they had no publication bias.

### 3.4. DFS (PCR)

**Figure 8.**
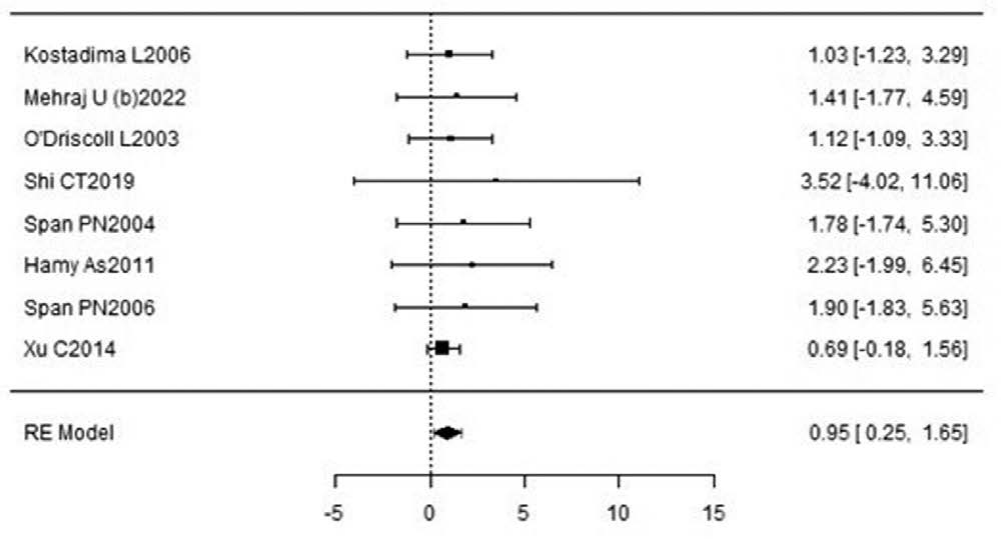
Forest plot for disease-free survival assessed by PCR method.

**Figure 9.**
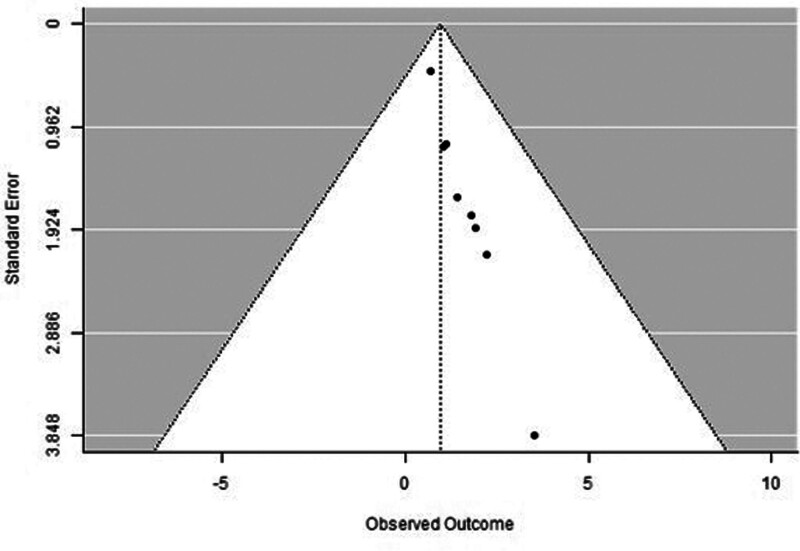
Funnel plot for disease-free survival assessed by PCR method.

A forest plot examining the association between survivin expression detected by PCR and DFS is shown in Figure 8. According to Figure 8, the study with the highest weight and consistency in the meta-analysis was Xu C (2014), while the study with the lowest weight and consistency was Shi CT (2019). It was shown that survivin expression evaluated by PCR method was associated with lower DFS (HR 0.95, 95% CI 0.25–1.65). The funnel plot evaluating the relationship between survivin expression detected by PCR and DFS is shown in Figure 9, where studies are concentrated in the middle part of the graph, indicating that the studies on DFS-PCR were moderately powered. Because the studies were within the confidence intervals, they had no publication bias.

### 3.5. Overall survival (IHC)

**Figure 10.**
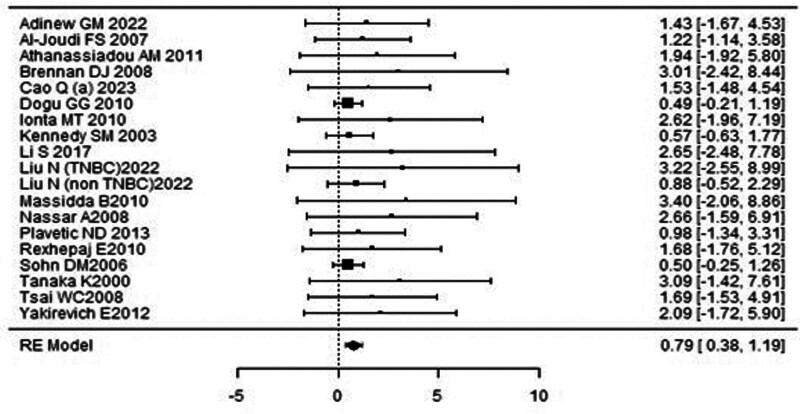
Forest plot for overall survival assessed by immunohistochemical method.

**Figure 11.**
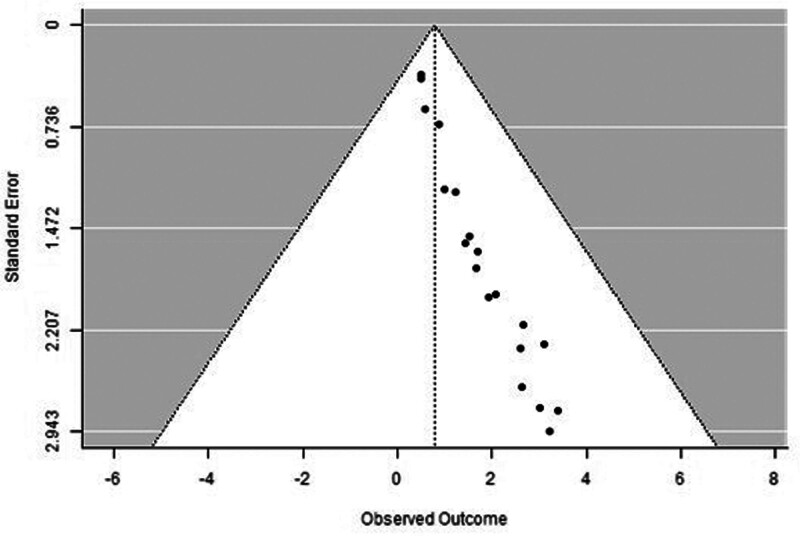
Funnel plot for overall survival assessed by immunohistochemical method.

A forest plot examining the association between survivin expression detected by IHC and OS is shown in Figure 10. According to Figure 10, the studies with the highest weight and consistency in the meta-analysis were Dogu GG (2010) and Sohn DM (2006) while the studies with the lowest weight and consistency were Brennan DJ (2008), Liu N (non-TNBC) (2022), and Massidda B (2010). It was shown that survivin expression evaluated by the IHC method was associated with lower DFS (HR 0.79, 95% CI 0.38–1.19). The funnel plot evaluating the relationship between survivin expression detected by IHC and OS is shown in Figure 11, where studies are concentrated in the lower part of the graph, indicating that the studies on OS IHC were weakly powered. Because the studies were within the confidence intervals, they had no publication bias.

### 3.6. Overall survival (PCR)

**Figure 12.**
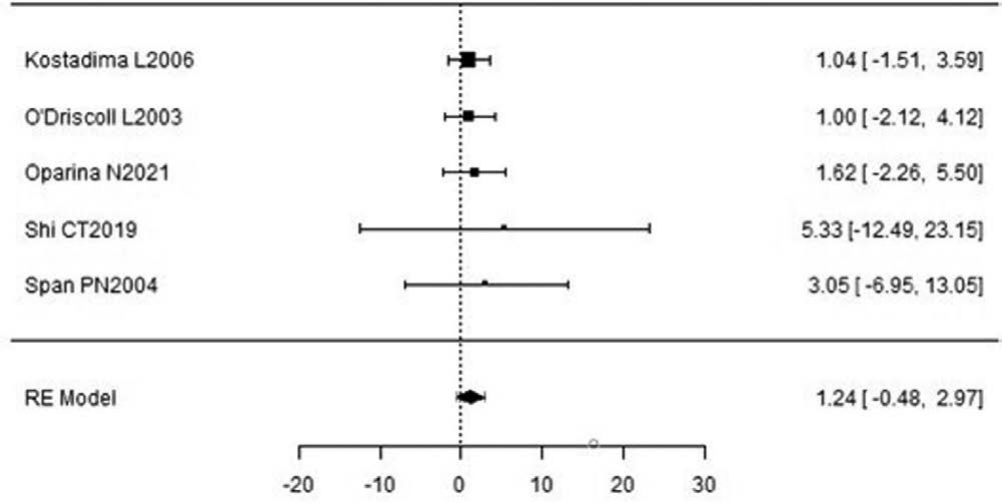
Forest plot for overall survival assessed by PCR method.

**Figure 13.**
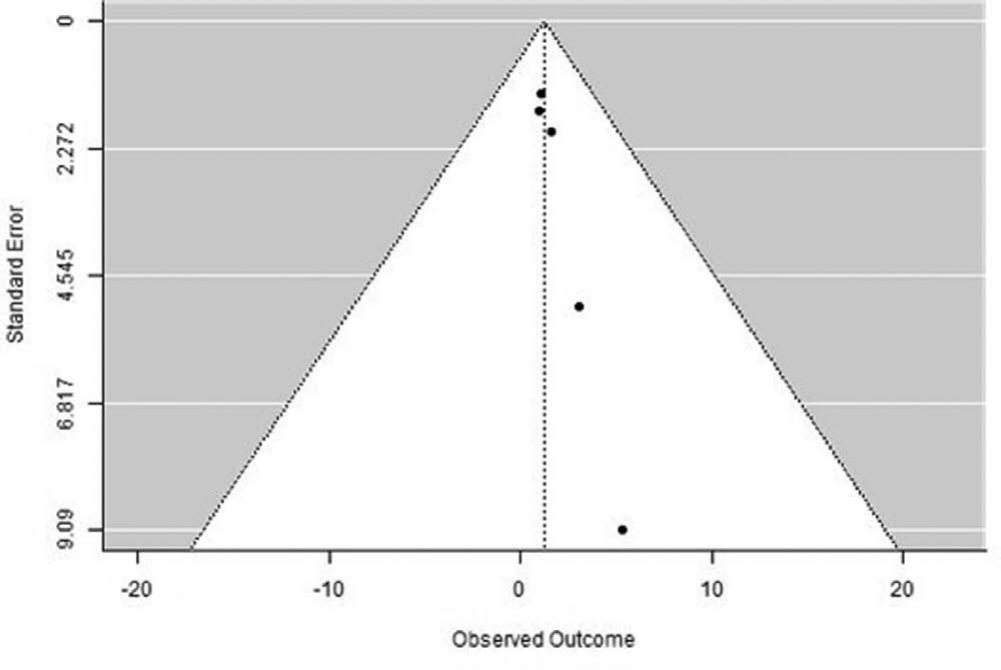
Funnel plot for overall survival assessed by PCR method.

A forest plot examining the association between survivin expression detected by PCR and OS is shown in Figure 12. According to Figure 12, the studies with the highest weight and consistency in the meta-analysis were Kostadima L (2006) and O’Driscoll L (2003) while the studies with the lowest weight and consistency were Shi CT (2019) and Span PN (2004). Survivin expression’s contribution to OS was limited, and the lack of statistically significant results in the random-effects model indicates caution in interpreting this subgroup (HR 1.24, 95% CI −0.48 to 2.97). The funnel plot evaluating the relationship between survivin expression detected by PCR and OS is shown in Figure 13, where studies are concentrated in the middle part of the graph, indicating that the studies on OS-PCR were moderately powered. Because the studies were within the confidence intervals, they had no publication bias.

Meta-analysis showed that survivin expression is a poor prognostic factor for both DFS and OS in breast cancer patients, with the effect of survivin expression on prognosis being independent of the method used to determine survivin expression.

## 4. Discussion

This meta-analysis evaluated the relationship between survivin expression and survival outcomes in patients with breast cancer. We conducted a meta-analysis of 24 studies evaluating the relationship between OS and survivin expression and 15 studies evaluating the relationship between DFS and survivin expression in breast cancer. Our findings indicated that survivin was a poor prognostic factor in both groups. The results remained consistent when categorized by the detection method, either IHC or PCR. However, the effect was limited in the study evaluating the association between survivin expression by PCR and OS. However, the random-effects model calculations suggested that the results of this subgroup meta-analysis may not be valid.

Tanaka et al analyzed survivin expression by IHC in 167 patients with stage I, II, and III breast cancer who underwent surgery. Survivin expression is not associated with OS.^[18]^ In a study by Al-Joudi et al, survivin expression was evaluated by IHC in 170 patients with breast cancer using a polyclonal antibody. They found no association between survivin expression and OS.^[23]^ In a study conducted by Cao et al, survivin expression evaluated by IHC had no significant effect on OS.^[24]^ Nassar et al included 37 breast cancer patients and evaluated survivin expression based on staining intensity and staining percentage, finding no association with OS and DFS.^[25]^ Gokoz Dogu et al evaluated survivin expression by IHC in 30 triple-negative breast cancer patients and found no significant relationship with DFS.^[26]^ In a study by O’Driscoll et al evaluating the survivin and survivin variants survivin-DEx3 and survivin-2B expression by PCR in 106 patients diagnosed with breast cancer, survivin expression was identified in 68% of samples and variants in 54.7%. They observed no association between survivin expression (including its variants) and DFS or OS.^[19]^ Kostadima et al qualitatively and quantitatively assessed survivin expression by PCR in 272 patients with stage II and III breast cancer who underwent surgery. They found that survivin expression was not associated with DFS or OS.^[27]^

Kennedy et al studied survivin expression by IHC in 293 patients with breast cancer, assessing both cytoplasmic and nuclear staining, with staining below 20% considered negative. Sixty percent of the patients were positive by IHC. They found that a lack of nuclear survivin expression was associated with shorter relapse-free survival, while cytoplasmic survivin expression was not associated with relapse-free survival. Furthermore, nuclear survivin expression has been linked to OS. Multivariate analysis showed that the relationship between nuclear survivin expression and survival persisted. Nuclear survivin expression is an independent risk factor for relapse-free survival and OS. Their results indicated that nuclear survivin expression above 20% decreased the risk of relapse and death compared to those without expression.^[20]^ Similar findings were observed in a study by Okada et al, which evaluated survivin expression in patients diagnosed with gastric cancer. They found that nuclear survivin expression was associated with a good prognosis, whereas cytoplasmic survivin expression was linked to poor prognosis.^[28]^ Survivin functions as both a chromosomal passenger protein in the nucleus and a cytoplasmic microtubule-associated protein. It is typically expressed during the G2/M phase of the cell cycle in a regulated manner and is associated with mitotic spindle microtubules during mitosis. It has been suggested that disrupting the interaction between survivin and microtubules may impair its antiapoptotic function, leading to increased caspase-3 activity during mitosis.^[29]^ Using a novel panel of monoclonal and polyclonal antibodies, Fortugno et al showed that survivin is present in different cellular locations. Nuclear and cytoplasmic survivin are immunohistochemically distinct and independently regulated during the cell cycle, and only cytosolic survivin is associates with p34cdc2. They found that phosphorylation of survivin by p34cdc2-cyclin B is necessary for inhibition of apoptosis, a requirement that may not apply to nuclear survivin.^[30]^ Oparina et al used PCR to show that survivin expression is associated with longer OS.^[31]^ Plavetic et al analyzed data from 188 patients out of 215 tumor samples using the tissue microarray method and reported that patients with 60% or more staining had longer DFS and longer OS.^[32]^ Athanassiadou et al studied 140 breast cancer patients, who underwent surgery and evaluated survivin expression by IHC using nuclear or cytoplasmic survivin staining. They found that patients with survivin expression had lower overall survival.^[33]^ Brennan et al used IHC and tissue microarray method to evaluate nuclear and cytoplasmic survivin expression by IHC in 102 breast cancer patients, who underwent surgery. They found that survivin expression was associated with lower survival.^[34]^ Ionta et al evaluated survivin expression using IHC in 53 patients diagnosed with breast cancer and found that it was associated with lower DFS and OS.^[35]^ Li et al evaluated survivin expression by IHC in 110 patients diagnosed with stage I-III breast cancer. Nuclear survivin expression is associated with lower OS and progression-free survival.^[36]^ Liu et al divided 142 breast cancer patients into triple-negative and non-triple-negative groups, assessing nuclear and cytoplasmic survivin expression by IHC. In the triple-negative group, survivin expression was correlated with lower DFS and OS, whereas in the non-triple-negative group, there was no association with survival, a finding that persisted in multivariate analysis.^[37]^ Massiada et al also demonstrated that survivin expression, assessed by IHC in breast cancer patients, was linked to lower OS.^[38]^ Rexhepaj et al studied survivin expression in 359 breast cancer patients and found that nuclear survivin expression was associated with lower OS.^[39]^ Sohn et al evaluated survivin expression by IHC in 80 breast cancer patients, noting that combined cytoplasmic and nuclear survivin expression showed no association with OS, but cytoplasmic survivin expression alone was associated with lower OS.^[40]^ Tsai et al evaluated survivin expression by IHC in invasive breast cancer, ductal carcinoma in situ, and normal breast tissue and found that high survivin expression in invasive breast cancer was associated with lower OS.^[41]^ Yakirevich et al examined survivin and acetylated survivin expression in 226 breast cancer patients, finding that survivin expression was associated with lower OS, while acetylated survivin had no significant relationship with OS. Multivariate analysis confirmed the relationship between survivin levels and survival.^[42]^ Kleinberg et al found that cytoplasmic survivin expression, as assessed by IHC, was associated with lower DFS in patients with breast cancer.^[43]^ Adinew et al found that higher survivin expression, as assessed by IHC, in triple-negative breast cancer patients was linked to lower DFS and OS.^[44]^ Span et al evaluated survivin expression using the quantitative PCR method and found that survivin mRNA concentrations in tumor tissue ranged from 10 to 14,000 copies. High survivin expression is associated with lower relapse-free survival and OS. Multivariate analysis confirmed that the relationship between survivin expression and survival remained unchanged, and survivin was a poor prognostic factor for survival.^[21]^ Shi et al included 150 patients with triple-negative breast cancer who underwent surgery and found through quantitative PCR that survivin expression was associated with lower DFS and OS.^[45]^ Yamashita et al evaluated survivin expression by quantitative PCR in breast cancer patients and found that high survivin expression correlated with lower DFS and OS.^[46]^ Hamy et al used PCR to evaluate survivin expression in stage 2 and stage 3 breast cancer patients receiving neoadjuvant chemotherapy and found that DFS was lower in the group with higher survivin expression.^[47]^ Span et al evaluated survivin and its variants (survivin-deltaEx3, survivin 2alfa, survivin-2beta, and survivin-3beta) in 275 breast cancer patients by PCR, finding that survivin, survivin 2alfa, and survivin 3beta were associated with relapse-free survival, a relationship that also persisted in multivariate analysis.^[48]^ Xu et al evaluated survivin expression using quantitative PCR in patients with stage I, II, and III breast cancer and found that high survivin expression was associated with lower DFS.^[49]^ Mehraj et al examined the relationship between survivin expression, and DFS in patients with breast cancer and showed that survivin expression correlated with lower DFS.^[50]^

The discrepancies in studies using PCR to evaluate the impact of survivin expression on prognosis in terms of OS and DFS may stem from differences in qualitative versus quantitative methods, patient groups (histological subtypes, clinicopathological prognostic factors), follow-up periods, disease stages, and unequal distribution of clinicopathological factors among the groups with high or low survivin expression.

Similarly, differences in studies using IHC may result from variations in antibodies, staining methods, evaluation techniques, lack of standardized positivity thresholds, patient stage heterogeneity, histological subgroup differences, follow-up variations, molecular subgroup differences, and survivin localizations within cells. Despite some studies indicating that survivin expression’s prognostic effect persists in multivariate analysis, these discrepancies complicate its use as a standalone prognostic marker.

The impact of survivin expression on prognosis has been widely studied in various cancers, including breast cancer. It is one of the most extensively investigated proteins in patients with cancer. Our meta-analysis indicated that increased survivin expression is generally associated with poor DFS and OS. However, some studies showed that the effect of survivin expression on survival was lost in multivariate analysis, suggesting that patient groups may not be equally distributed regarding known clinicopathological prognostic factors when grouped by survivin expression levels. Additionally, the presence of studies indicating that survivin expression has no effect on survival or is even associated with favorable survival suggests that patient groups may differ in terms of prognostic factors. The localization of survivin within the cell (nuclear or cytoplasmic) may contribute to these varying results in terms of prognosis. Our meta-analysis revealed that the IHC analysis and PCR method subgroups had prognostic effects on DFS and OS. However, using survivin expression as a reliable prognostic factor is challenging because of the difficulty in assessing it via PCR and the lack of standardization in IHC evaluations.

## 5. Conclusion

In conclusion, survivin expression is significantly elevated in breast cancer tissues compared to that in normal tissues and is an important prognostic marker. Increased survivin expression is generally associated with poor DFS and OS, affecting treatment response and prognosis. While survivin may be considered during treatment planning or as a treatment target, contradictory results and a lack of standardization in evaluation methods suggest that it should not be used alone as a prognostic marker. Therefore, other clinicopathological prognostic markers should be considered.

## 6. Limitations of the study

Since the studies included in the meta-analysis were retrospectively designed, involving patient groups with various histological subtypes and disease stages without standardized evaluation methods, the overall effect size of the meta-analysis was considered moderate.

## Author contributions

**Conceptualization:** Betul Bolat Kucukzeybek, Yasemin Basbinar, Mustafa Oktay Tarhan.

**Data curation:** Betul Bolat Kucukzeybek, Yuksel Kucukzeybek, Cigdem Dinckal.

**Formal analysis:** Betul Bolat Kucukzeybek, Yuksel Kucukzeybek, Yasemin Basbinar, Hulya Ellidokuz, Cigdem Dinckal.

**Funding acquisition:** Betul Bolat Kucukzeybek, Yasemin Basbinar.

**Investigation:** Betul Bolat Kucukzeybek, Yuksel Kucukzeybek, Mustafa Oktay Tarhan.

**Methodology:** Betul Bolat Kucukzeybek, Yasemin Basbinar, Hulya Ellidokuz, Mustafa Agah Tekindal.

**Project administration:** Betul Bolat Kucukzeybek, Yuksel Kucukzeybek, Yasemin Basbinar.

**Resources:** Betul Bolat Kucukzeybek, Cigdem Dinckal.

**Software:** Hulya Ellidokuz, Mustafa Agah Tekindal.

**Supervision:** Yasemin Basbinar, Mustafa Oktay Tarhan.

**Writing – original draft:** Betul Bolat Kucukzeybek, Yuksel Kucukzeybek, Mustafa Oktay Tarhan.

**Writing – review & editing:** Betul Bolat Kucukzeybek.
